# Exploring the mechanisms of Cangfu Daotan decoction in homotherapy for heteropathy of polycystic ovary syndrome, insulin resistance, infertility, and obesity

**DOI:** 10.1097/MD.0000000000048205

**Published:** 2026-04-17

**Authors:** Hongjing Chen, Li Deng, Haobo Chen, Binbin Cai

**Affiliations:** a Wenzhou Hospital of Integrated Traditional Chinese and Western Medicine Affiliated to Zhejiang Chinese Medical University, Wenzhou, Zhejiang, China; b College of Integration of Traditional Chinese Medicine and Western Medicine, Southwest Medical University, Luzhou, Sichuan, China.

**Keywords:** Cangfu Daotan decoction, insulin resistance, network pharmacology, obesity and infertility, polycystic ovary syndrome

## Abstract

This study aims to explore the mechanism of Cangfu Daotan decoction (CFDT) in treating polycystic ovary syndrome, insulin resistance, obesity, and infertility with homotherapy for heteropathy based on network pharmacology methods. The active components and corresponding protein targets of CFDT were identified through a systematic screening of the Traditional Chinese Medicine Systems Pharmacology database, while disease-associated targets were retrieved from OMIM, Genecards, and DrugBank databases. Common targets were derived from Venn analysis and utilized to construct a protein–protein interaction network via STRING and Cytoscape 3.8.0, through which core targets were identified. Gene Ontology and Kyoto Encyclopedia of Genes and Genomes pathway assessment were subsequently performed on these key targets using R 4.1.1. Molecular docking simulations were finally conducted to evaluate binding interactions between pivotal bioactive compounds and the identified core targets. A total of 151 active ingredients, 238 drug targets and 2722 disease targets were screened. Among them, quercetin, kaempferol, luteolin, and wogonin are the main active ingredients. TP53, AKT1, STAT3, IL6, and HSP90AA1 are the core therapeutic targets. Kyoto Encyclopedia of Genes and Genomes pathway enrichment analysis screened 178 pathways, including lipid and atherosclerosis and advanced glycation end product (AGE)-receptor for AGE signal pathways in diabetes complications. Gene Ontology functional enrichment analysis yielded 2423 Gene Ontology entries, mainly involving biological processes including heterologous stimulation, lipopolysaccharide response, oxidative stress, glandular development, as well as cellular composition including membrane rafts and vesicles, and molecular functions including DNA binding transcription factor binding and cytokine activity. Molecular docking shows that the active ingredients of CFDT have good affinity for core disease targets. Molecular docking confirmed strong binding affinity between key compounds and targets. It is preliminarily revealed that the main active ingredients of CFDT are quercetin, kaempferol, luteolin and wogonin, which may improve polycystic ovary syndrome, insulin resistance, obesity, and infertility by regulating lipid and atherosclerosis and AGE-receptor for AGE signal pathways in diabetes complications.

## 1. Introduction

Polycystic ovary syndrome (PCOS) is a multifactorial, systemic and inflammatory condition that affects women of reproductive age.^[1]^ It is characterized by endocrine abnormalities, metabolic dysregulation and reproductive dysfunction. Common clinical manifestations of PCOS include menstrual irregularities, ovulatory dysfunction, hyperandrogenism, insulin resistance (IR) and obesity. Long-term complications may involve infertility, endometrial hyperplasia, endometrial cancer, type 2 diabetes, hyperlipidemia, and cardiovascular disease. PCOS has become an important cause of infertility among women today. The global prevalence of PCOS was estimated to range between 6% and 20%.^[2]^ Recent study has indicated that more than 90% of individuals with anovulatory infertility are also affected by PCOS.^[3]^

The pathogenesis of PCOS remains incompletely understood. Clinical observations have indicated that PCOS is closely associated with IR, obesity and infertility.^[4–8]^ Patients with PCOS often exhibit abnormalities in glucose and lipid metabolism as well as disrupted androgen levels.^[9–11]^ IR plays a vital role in obesity, particularly among PCOS patients with abdominal obesity.^[12,13]^ An uneven distribution of body fat frequently contributes to metabolic dysregulation, while obesity itself can aggravate hyperinsulinemia.^[14,15]^ Moreover, elevated levels of circulating androgens and insulin often result in ovarian impairment, thereby causing infertility.^[16–18]^ These 4 diseases interact with each other to form a vicious cycle that perpetuates and exacerbates the progression of PCOS.

Traditional Chinese Medicine (TCM) has a long history in treating the complications of PCOS.^[19,20]^ In TCM, the occurrence of PCOS, IR, obesity, and infertility are often considered to be related to spleen deficiency and phlegm-dampness, which manifest clinically as menstrual irregularities, ovulatory dysfunction, and systemic metabolic disturbances.^[21]^ As recorded in *Su Wen*, “fluids enter to the stomach, and transported upward to the spleen.” In TCM theory, dysfunction in the spleen and stomach circulation would lead to the retention of phlegm-dampness and qi blockage, which were considered the key mechanisms in the pathological progression of PCOS.^[22]^ The *Danxi Xinfa* further pointed out that infertility is primarily caused by “excessive phlegm-dampness blocking the uterus.” Spleen deficiency results in excessive dampness and the stagnation of phlegm-dampness, which obstructs the normal movement of qi and blood.^[23,24]^ These TCM concepts may correspond to metabolic dysregulation and hormonal imbalance observed in PCOS from modern medical perspective.^[22,23]^

The method of “homotherapy for heteropathy” is one of the main TCM principles, where unrelated illnesses show the same underlying imbalances, like how PCOS, IR, infertility, and obesity all stem from “Qi deficiency and phlegm accumulation,” which could ultimately allow these conditions to receive identical treatment. This approach targets the shared pattern (the cause of diseases called “*zheng*” in TCM) rather than surface symptoms, indicating a single herbal formula could effectively address multiple diseases when they share the same pattern. Cangfu Daotan decoction (CFDT), a classic TCM formula recorded in the literature *Yeshi Nvke*, has been commonly used to treat menstrual disorders in women resulting from spleen deficiency and phlegm accumulation. Its functions include dry dampness and transform phlegm, regulating qi and unblock collaterals. Recent studies have indicated that CFDT can improve follicular development and alleviate IR and obesity in PCOS with phlegm-dampness syndrome.^[24–27]^ This study aims to employ network pharmacology to identify the potential mechanisms of CFDT in homotherapy for heteropathy of PCOS, insulin resistance, infertility and obesity.

## 2. Materials and methods

### 2.1. Screening active ingredients of CFDT

The active components of CFDT were identified using the Traditional Chinese Medicine Systems Pharmacology (TCMSP, https://www.91tcmsp.com) database. The active ingredients of *Arisaematis Rhizoma* (Tiannanxing, 6g), *Cyperi Rhizom* (Xiangfu, 10g), *Atractylodes Lancea* (Cangzhu, 15g), *Poria* (Fuling, 15g), *Aurantii Fructus* (Zhiqiao, 12g), *Citrus Reticulata* (Chenpi, 10g), and *Licorice* (Gancao, 5g) were screened in TCMSP based on related parameters including oral bioavailability (≥ 30%) and drug-likeness ( ≥ 0.18).

### 2.2. Target acquisition

The regulatory targets of active ingredients Tiannanxing, Xiangfu, Cangzhu, Fuling, Zhiqiao, Chenpi, and Gancao were download from the TCMSP database, perform gene name calibration in the Uniprot database, and obtain the potential gene targets regulated by the active ingredients. At the same time, the Isomeric simplified molecular input line entry system sequence numbers of all active ingredients were obtained from the organic small molecule bioactive database PubChem, and imported into the SwissTsgetPrediction target prediction website. The screening criteria were based on the possibility > 0.5, and the component targets obtained from the 2 databases were merged to remove duplicate targets, ultimately obtaining the targets regulated by the active ingredients.

### 2.3. Screening targets of PCOS, IR, infertility, and obesity

Disease-related genes were identified by searching the GeneCards (https://www.genecards.org), DrugBank (https://go.drugbank.com) and OMIM (https://www.omim.org) databases. Then the intersection targets of the 4 diseases were screened.

### 2.4. Screening for intersection targets between CFDT and diseases

The intersection targets between CFDT and the 4 diseases (PCOS, IR, infertility, and obesity) were visualized employing jvenn (https://jvenn.toulouse.inrae.fr/app/example.html) to create the Venn diagram. Then the hypergeometric test was performed using phyper function in R 4.1.1 (R Core Team, Vienna, Austria), referring to the number of experimentally validated human disease-associated genes recorded in the UniProt (https://www.uniprot.org/) database, the number of total human genes was set as the background (N = 205,228), and the significance of the overlap was calculated.

### 2.5. Construct drug-component-intersection targets network

The active components of CFDT and their corresponding target genes were imported into Cytoscape 3.8.0 (Cytoscape Consortium, San Diego), to construct a compound–target interaction network. Each node represented an active compound, and each edge represented its interaction with the corresponding target. Network topology analysis was conducted using the network analyzer plugin in Cytoscape 3.8.0 and calculate the degree value of each node. The degree values reflect the number of target genes connected to each compound, and compounds with degree values >20 were identified as the core active components of CFDT.

### 2.6. Construction of protein–protein intersection (PPI)

The intersection targets of CFDT and diseases were imported to the STRING (STRING Consortium, Zurich, Switzerland), database (https://cn.string-db.org) to construct the protein-protein network. Then the resulting network was analyzed in Cytoscape 3.8.0 software to obtain core targets based on following topological parameters, including degree centrality, betweenness centrality, closeness centrality, eigenvector centrality, local average connectivity, and network centrality. The core target was finally obtained by 3 times screening according to the median. Core targets were screened through 3 iterative rounds, in which only nodes with all 6 parameter values greater than their respective medians were retained at each round.

### 2.7. Enrichment analysis of intersection targets

The ClusterProfiler package were used to perform Gene Ontology (GO) and Kyoto Encyclopedia of Genes and Genomes (KEGG) enrichment analyses in R 4.1.1, and the enrichment results were filtered based on *P* value < .05 and *q* value < .05. The top 30 significantly enriched terms of GO and KEGG were visualized eventually.

### 2.8. Molecular docking

The 2D chemical structures of the key active ingredients in CFDT were downloaded from the PubChem (https://pubchem.ncbi.nlm.nih.gov/) and converted into 3D structures by Chem3D software. The structures were then saved as ligand files in mol2 format.

The 3D structures of the core target proteins were downloaded from the PDB database (https://www.rcsb.org/). The proteins underwent preprocessing steps in PyMOL version 2.6 (Schrödinger, Inc., New York), and AutoDockTools version 1.5.6 software (Scripps Research Institute, La Jolla), including water molecules removal, hydrogenation and small molecule elimination. The protein receptor files were exported in PDB format.

Molecular docking was performed in AutoDock Vina version 1.1.2 (Scripps Research Institute, La Jolla), where protein receptors were docked with the small molecule ligand. The grid box was set to cover the entire receptor protein, and each ligand–receptor pair was docked 20 times. The minimum binding energies were calculated and visualized with the PyMOL version 2.6. Core active ingredients were identified based on a binding energy threshold of ≤ −5.0 kcal/mol. These ingredients were further validated through docking with the core targets to confirm their interactions.

### 2.9. Statistical analysis

The hypergeometric test of the intersection targets was carried out by using the phyper function of R 4.1.1, and the statistical significance was *P* < .05.

## 3. Results

### 3.1. Screening of CFDT active ingredients and targets

After screened through TCMSP database by oral bioavailability ≥ 30% and drug-likeness ≥ 0.18, a total of 151 active ingredients were acquired, including 7 active compounds from Dannanxing, 9 active compounds from Cangzhu, 15 active compounds from Fuling, 18 active compounds from Xiangfu, 5 active compounds from Zhiqiao, 5 active compounds from Chenpi and 92 active compounds from Gancao. Among these 151 active ingredients, 238 potential targets were identified.

### 3.2. Intersection target screening

**Figure 1.**
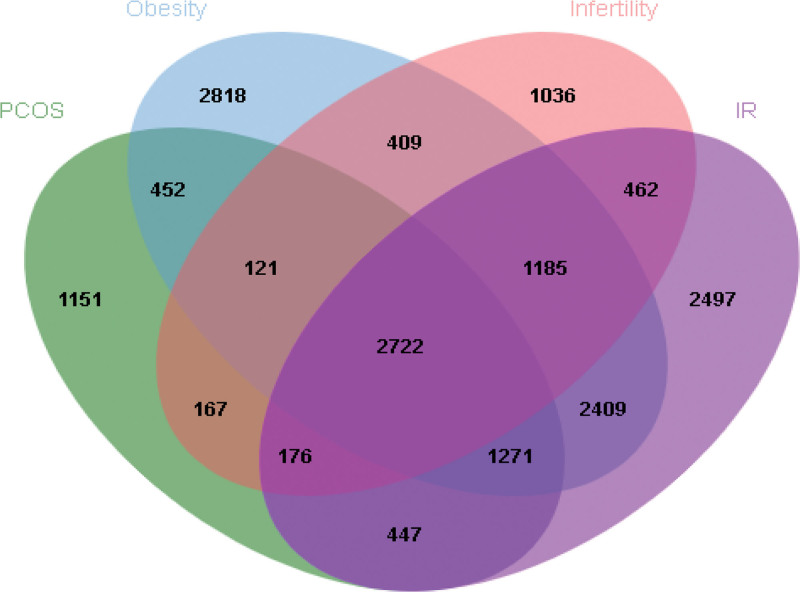
The genes of PCOS, IR, infertility and obesity. IR = insulin resistance, PCOS = polycystic ovary syndrome.

**Figure 2.**
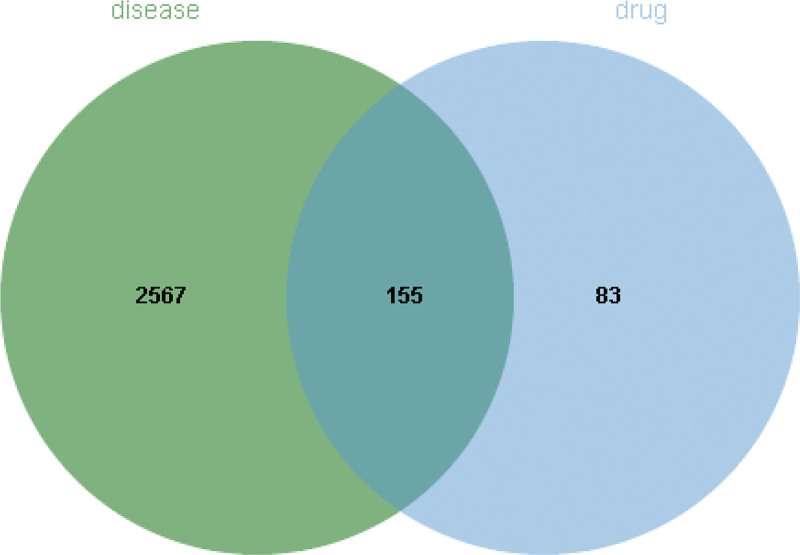
“Drug-disease” intersection targets.

The genes for 4 diseases were obtained from OMIM, Genecards, and DrugBank, and a total of 2722 targets were collected (Fig. 1). After the of drug targets and disease genes, 155 targets were obtained (Fig. 2), which were considered as potential therapeutic targets. Furthermore, a hypergeometric test performed in R 4.1.1 confirmed that a statistically significant enrichment of shared targets between CFDT and the 4 diseases (*P* < .001).

### 3.3. Construct “drug-ingredient-targets” network diagram

**Figure 3.**
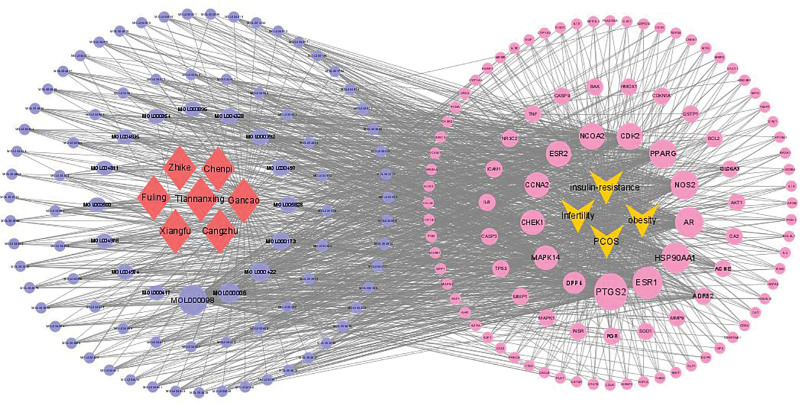
Drug-ingredient-target network of CFDT. CFDT = Cangfu Daotan decoction, PCOS = polycystic ovary syndrome.

The active compounds and intersection targets of CFDT were imported into Cytoscape 3.8.0 and visualized by this software (Fig. 3). The analysis revealed that quercetin, luteolin, kaempferol, and wogonin were the top 4 key active components of CFDT (Table 1).

**Table 1:** Top 4 active ingredients of CFDT.

Molecule id	Active components	Degree	Related herbs
MOL000098	Quercetin	74	Xiangfu, Gancao
MOL000006	Luteolin	35	Xiangfu
MOL000422	Kaempferol	28	Xiangfu, Gancao
MOL000173	Wogonin	23	Cangzhu

CFDT = Cangfu Daotan decoction.

### 3.4. PPI network of intersection targets of CFDT and diseases

**Figure 4.**
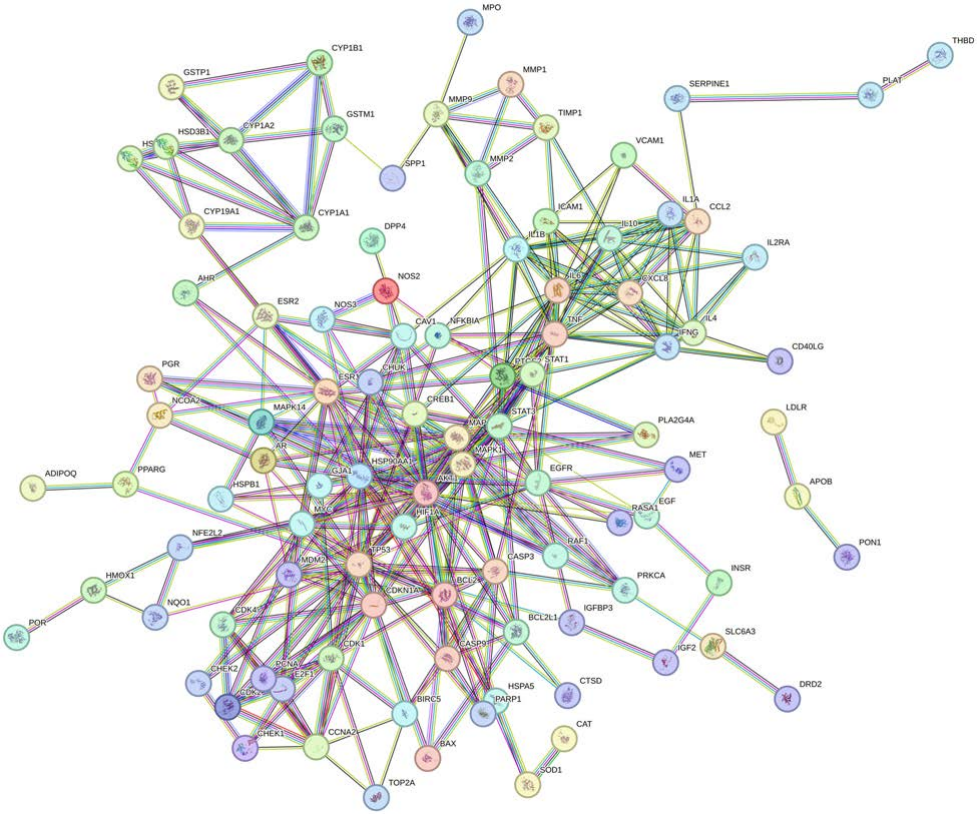
PPI network of intersection targets. PPI = protein–protein intersection.

**Figure 5.**
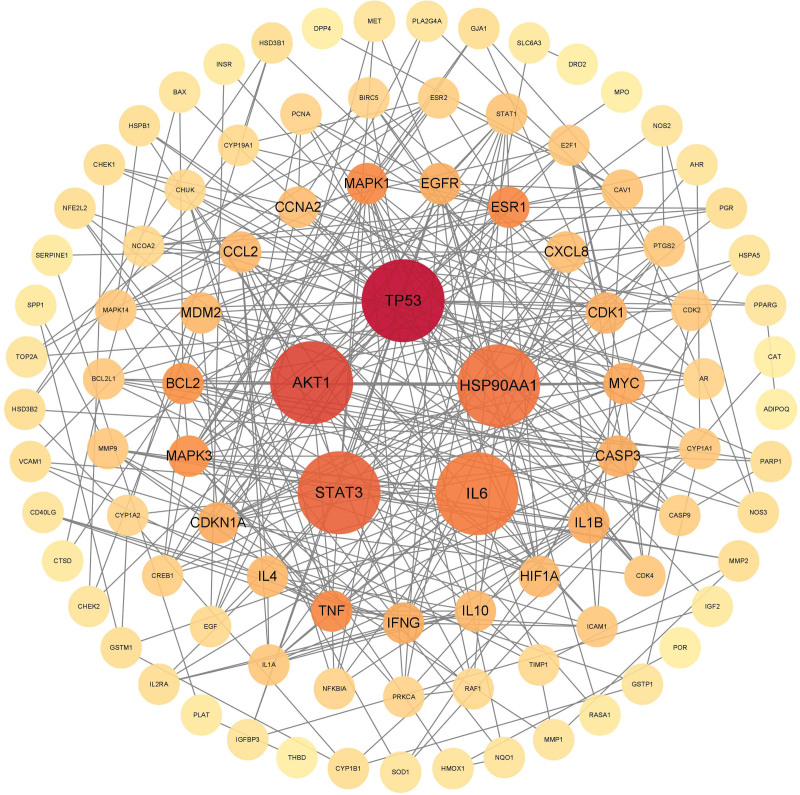
Core genes PPI network. PPI = protein–protein intersection.

The 155 intersection targets were imported into the STRING platform to build the PPI network (Fig. 4). PPI network comprised 155 nodes and 353 edges, with an average node degree of 6.79. Core targets were then identified using Cytoscape 3.8.0, and TP53, AKT1, STAT3, IL6, and HSP90AA1 were showed the highest score (Fig. 5).

### 3.5. GO and KEGG enrichment analysis

**Figure 6.**
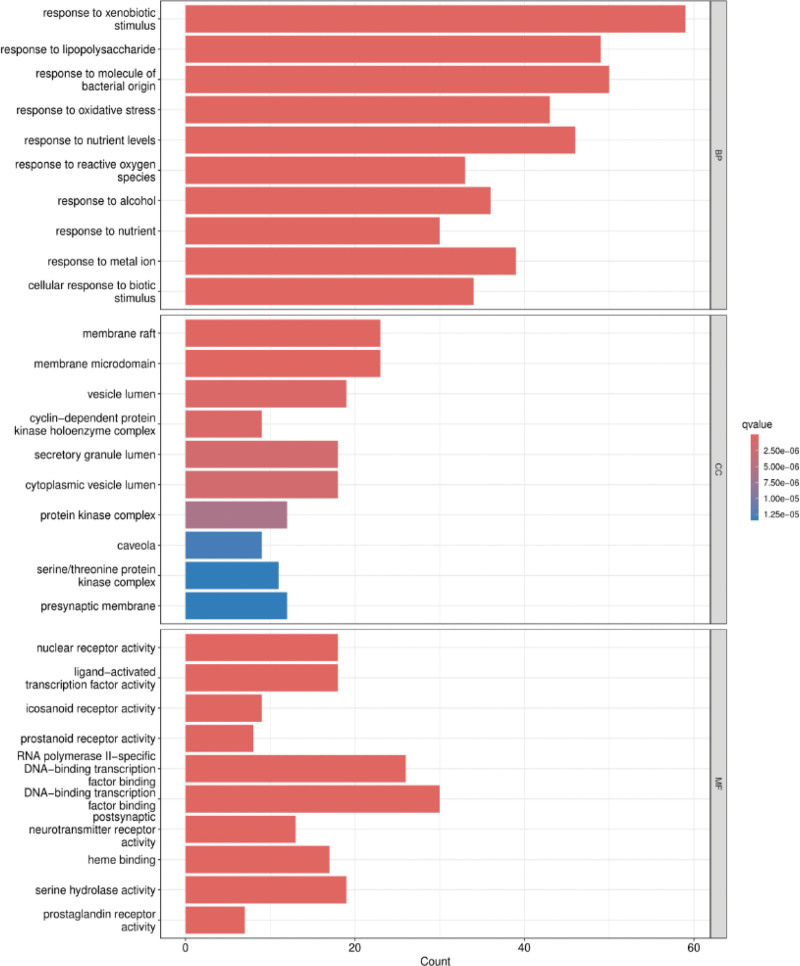
GO enrichment analysis. GO = gene ontology.

R 4.1.1 was utilized to perform GO function enrichment analysis of the 155 key targets, and 2423 entries were obtained (*P* < .05). These included a total of 2192 biological processes, primarily related to xenobiotic stimulation, lipopolysaccharide reaction, oxidative stress and gland development. There are 69 cellular components, with key locations including membrane rafts, membrane microdomains, vesicle cavities. Additionally, 161 molecular functions were identified, such as DNA − binding transcription factor binding, RNA polymerase II − specific DNA − binding transcription factor binding and nuclear receptor activity. The top 10 enriched terms in each category were visualized in Figure 6.

**Figure 7.**
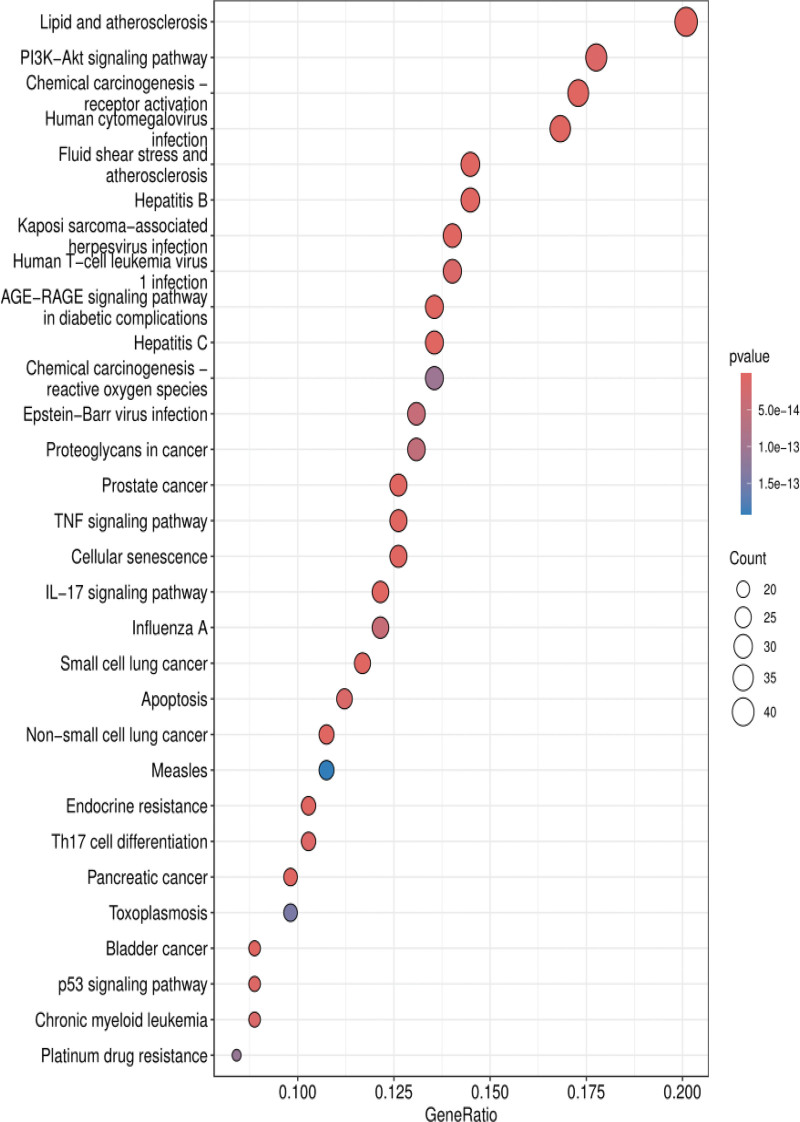
KEGG enrichment analysis. KEGG = Kyoto Encyclopedia of Genes and Genomes.

One-hundred and seventy-eight signaling pathways (*P* < .05) were obtained in KEGG pathway enrichment analysis, with the primarily pathway including lipid and atherosclerosis signaling pathway, advanced glycation end product-receptor for AGE (AGE-RAGE) signaling pathway in diabetic complications. The top 30 with the smallest *P* value pathways were visualized (Fig. 7). The analysis revealed that core targets included TP53, AKT1, HSP90AA1, and TNF were significantly enriched in these pathways.

### 3.6. Molecular docking

**Figure 8.**
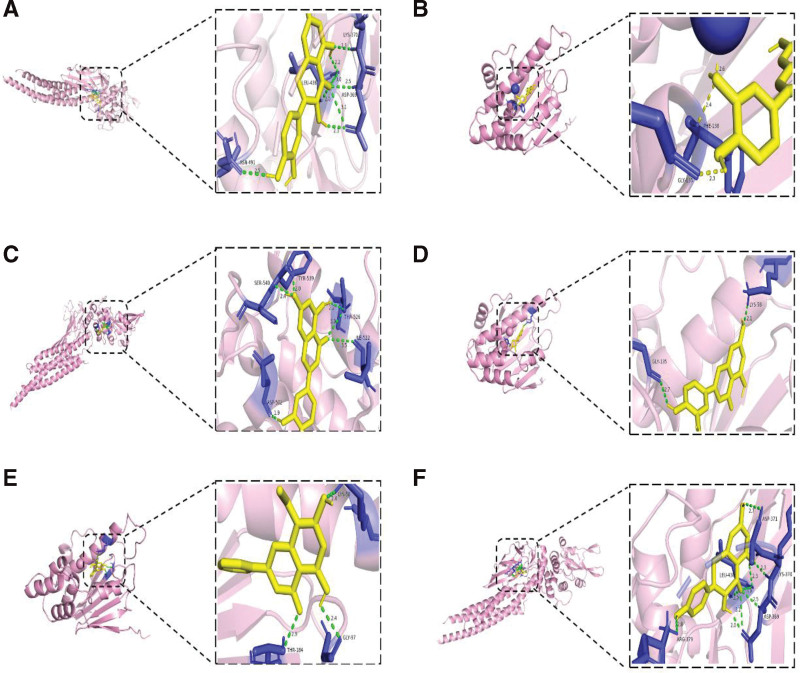
Conformation diagram of molecular docking between active components of CFDT and core target molecules. (A) Quercetin – STAT3, (B) quercetin – HSP90AA1, (C) luteolin – STAT3, (D) lueteolin – HSP90AA1, (E) wogonin – HSP90AA1, (F) kaempferol – STAT3.

The molecular docking results demonstrated that the key active components of CFDT exhibited a strong affinity for core targets of 4 diseases. Among these components, quercetin, kaempferol, wogonin, and luteolin showed robust binding structures with all the critical targets. The docking results with docking energy <−7.5 kcal/mol were visualized (Fig. 8). The 3 compounds were identified as the core active ingredients of CFDT (Table 2).

**Table 2:** Affinity value of CFDT main ligand and receptor.

Protein affinity (kcal/mol) compound	TP53	AKT1	STAT3	HSP90AA1	IL6
Quercetin	−6.2	−6.3	−8.2	−7.6	−7.2
Luteolin	−6.3.	−6.4	−8.0	−7.6	−7.2
Wogonin	−5.6	−6.1	−7.3	−7.9	−6.4
Kaempferol	−5.8	−6.1	−8.0	−7.0	−6.8

CFDT = Cangfu Daotan decoction.

## 4. Discussion

Modern studies have demonstrated that PCOS often coexists with obesity, IR, and infertility, which interacting through a network of inflammatory and metabolic disturbances. In TCM theory, the pathogenesis of obesity-related PCOS is closely associated with dysfunction of the liver, spleen and kidney, with the pattern of “phlegm-dampness retention and impaired spleen transportation” playing a central role. Multiple studies have indicated that CFDT can markedly restore a normal menstrual cycle, regulate hormone levels and improve ovarian function in patients with obesity-associated PCOS.^[28,29]^ As recorded in *Danxi Xinfa,* “to treat phlegm, 1 must first regulate qi; when qi flows freely, the body fluids follow.” The formula CFDT was designed to regulate qi and resolve phlegm, aligning with the holistic principle of homotherapy for heteropathy of PCOS, IR, obesity, and infertility.

Within the prescription, the herbs Xiangfu and Cangzhu work synergistically, 1 dispersing qi stagnation and the other drying dampness. *Bencao Gangmu* noted that Xiangfu “benefits the sanjiao, relieves the qi stagnation and eliminates phleegm,” while as recorded in *Shennong Bencaojing* Cangzhu “treats wind-cold-damp obstruction.” Zhiqiao and Chenpi further regulate qi and harmonize the zhongjiao, reinforcing Xiangfu effect in relieving stagnation. As *Yaopin Huayi* stated: “Chenpi regulates qi and resolves phlegm, Zhiqiao regulates qi and transform indigestion,” highlighting the therapeutic principle that “when qi is regulated, phlegm naturally dissolves.” Supporting herbs include Fuling, which strengthens the spleen and transforms dampness. As recorded in *Shibuzhai Yishu, “*Fuling is the chief herb for treating phlegm.” Dan Nanxing could clear heat and resolve phlegm. Finally, Gancao harmonizes the formula and tonifies qi.

In this study, it was found that the key active components of CFDT were quercetin, luteolin, kaempferol, and wogonin. Quercetin, as a natural flavonoid, has many pharmacological effects such as antihyperlipidemia, antioxidation, antiinflammation and antihyperglycemia.^[30–33]^ Quercetin could comprehensively regulate the glucose and lipid metabolism, sex hormone level, and ovarian oxidative damage in PCOS patients.^[34–36]^ Meta-analysis of several PCOS studies showed that quercetin could treat PCOS with IR by regulating metabolic pathways such as blood sugar and lipid, and reducing testosterone and luteinizing hormone levels.^[37]^ In addition, quercetin also could regulate the expression of inflammatory cytokines such as IL-1β, IL-6, and TNF-α in ovarian tissues, thereby alleviating ovarian damage and improving infertility.^[38,39]^ Luteolin is a natural flavonoid with potential pharmacological activities of protecting ovaries and improving PCOS.^[40,41]^ Luteolin could regulate the level of sex hormones in PCOS rats, and improve ovarian function through metabolic pathway and antioxidation.^[42]^ In addition, luteolin could effectively inhibit the expression of aromatase and improve the hormone synthesis function of human ovarian granulosa cells.^[43]^ Kaempferol, as a flavonoid, has pharmacological activities including promoting fat consumption^[44,45]^ and improving insulin sensitivity.^[46,47]^ Scientific studies have shown that kaempferol could improve the uterine microenvironment by regulating the level of reproductive hormones through the intestinal-uterine axis^[48]^ and it also has the benefits of reducing uterine inflammation, maintaining ovarian morphology and promoting follicular development.^[49–52]^ Wogonin has antitumor,^[53]^ antithrombotic^[54]^ and other activities. A research has shown that wogonin could decrease blood sugar by increasing glucose uptake in cells through the AKT/GLUT4 pathway, thus improving insulin resistance,^[55]^ and improving obesity-induced lipid metabolism disorder by inhibiting cell apoptosis and inactivating the IL-17 signaling pathway.^[56]^ These pharmacological findings provide a mechanism explanation of the TCM concept of “resolving phlegm and regulating qi,” suggesting that CFDT may alleviate metabolic and reproductive abnormalities in PCOS through multi-target regulation of inflammation and oxidative stress.

According to the PPI analysis, TP53, AKT1, STAT3, IL6, and HSP90AA1 were identified as the key targets of CFDT. These proteins may play important roles in oxidative stress, inflammation, and metabolic regulation and other related pathophysiological processes relevant to obesity-associated PCOS. TP53 is an important factor in cell cycle regulation and plays a central role in cellular stress response, metabolic disorder and tumorigenesis.^[57]^ The down-regulation of TP53 may lead to sex hormone imbalance and ovarian oxidative stress through TIGAR-mediated Nrf2/OH-1 pathway, thus aggravating PCOS.^[58]^ Additionally, TP53 could also induce glycolysis and apoptosis regulators, and inhibit oxidative stress and apoptosis by activating Nrf2/OH-1 pathway in PCOS. Moreover, a recent study has shown that overexpression of growth differentiation factor 8 could damage the metabolic function of ovarian granulosa cells, and TP53 played a key role in this process.^[59–61]^ AKT1 is the key protein that regulates ovarian function and insulin signal transduction.^[62–64]^ The high expression of AKT1 is related to hyperandrogenism of PCOS,^[65]^ while downregulation of AKT1 expression could significantly improve IR and PCOS.^[66–68]^ STAT3 is a transcription factor and its activation is related to chronic inflammation and IR accompanied by obesity.^[69,70]^ Studies have shown that regulate the activity of STAT3 could reduce ovarian granulosa cell death in PCOS.^[71,72]^ IL-6 is a proinflammatory factor, and the increase of IL-6 will lead to tissue damage. Studies have shown that IL-6-induced chronic inflammatory infiltration was related to obesity, IR and the severity of ovulation disorder in PCOS patients.^[73–76]^ HSP90AA1 protein is involved in cell stress and signal transduction, and related to IR and fat deposition. Studies have shown that the HSP90AA1 expression serves as an important marker of obesity and infertility^[77,78]^ and it is also involved in the activation of metabolism-related inflammation.^[79]^

GO enrichment analysis shows that it is mainly enriched in biological processes such as oxidative stress and gland development, cellular components such as membrane rafts, membrane domains and vesicles, and molecular functions such as DNA-binding transcription factor binding, RNA–DNA−-binding transcription factor binding, cytokine activity and kinase regulatory activity. KEGG enrichment analysis revealed that CFDT has the potential regulatory effect on lipid metabolism, atherosclerosis and AGE-RAGE signaling pathway. AGE-RAGE signaling pathway is associated with diabetes and its complications. Studies have shown that the imbalance of AGE-RAGE signal could change the metabolic activity of follicular cells and exacerbates oxidative damage, thus affecting the ovarian development.^[80–82]^ The abnormal activation of AGE-RAGE also aggravates hyperandrogenism, IR and ovulation dysfunction of PCOS.^[81,83,84]^ Oxidative stress would aggravate the damage of ovarian granulosa cells in PCOS, especially in patients with obesity. Inflammation and oxidative stress would seriously affect the development of follicular cells,^[85–87]^ and oxidative stress could also activate the AGE-RAGE pathway to form the vicious circle from hyperandrogenism to PCOS.^[88]^ Lipid and atherosclerosis signaling pathways are related to PCOS and obesity, and influenced by glucose and lipid metabolism.^[89–91]^ Lipid deposition and atherosclerosis in arteries play an important role in the formation of PCOS-related cardiovascular complications. Moreover, IR and lipid metabolism also involved in this process.^[92–94]^ Clinical evidence showed that CFDT could effectively regulate the lipid metabolism level of PCOS patients with obesity.^[27,95]^ The further results of molecular docking showed that the key active components of CFDT have good affinity with the core targets of 4 diseases, supporting the network pharmacology findings and suggests that CFDT may act on PCOS, IR, obesity, and infertility through the above mechanisms. Collectively, these findings indicate that CFDT may exert synergistic effects on endocrine, metabolic, and inflammatory pathways, thereby restoring ovarian function and metabolic homeostasis in PCOS.

## 5. Limitations

This study has several limitations. First, all analyses were conducted entirely in silico using network pharmacology and molecular docking approaches, which cannot fully capture the complexity of biological systems in vivo. Second, the target information was derived from currently available databases, which may be incomplete or biased and therefore may not fully reflect the actual pharmacological effects of the drugs or the true pathological mechanisms of diseases. Consequently, further in vitro and in vivo studies are required to validate the predicted targets and mechanisms.

## 6. Conclusion

Overall, this study revealed the mechanism of CFDT in treating PCOS, IR, infertility, and obesity through network pharmacological analysis and molecular docking. It is found that quercetin, kaempferol, and kaempferol were the key active components and they could homotherapy for heteropathy of PCOS, IR, infertility, and obesity by regulating multiple targets such as TP53, AKT1, and IL-6. The action pathways including AGE-RAGE, lipid metabolism and atherosclerosis pathway, which may provide the theoretical basis for its clinical application. However, future experimental investigations should be conducted to validate these results, so as to better play the clinical efficacy of CFDT in metabolic diseases.

## Author contributions

**Conceptualization:** Hongjing Chen, Binbin Cai.

**Data curation:** Hongjing Chen.

**Formal analysis:** Hongjing Chen.

**Funding acquisition:** Binbin Cai.

**Investigation:** Haobo Chen.

**Methodology:** Li Deng.

**Project administration:** Binbin Cai.

**Software:** Hongjing Chen.

**Supervision:** Haobo Chen.

**Validation:** Hongjing Chen.

**Visualization:** Hongjing Chen, Li Deng.

**Writing – original draft:** Hongjing Chen, Li Deng.

**Writing – review & editing:** Hongjing Chen, Li Deng.
